# Hepatectomy for massive hepatic necrosis after transcatheter arterial embolization hemostasis for hepatic hemorrhage following hepatic trauma: A case report

**DOI:** 10.1002/ccr3.7888

**Published:** 2023-09-18

**Authors:** Takehiko Hanaki, Naruo Tokuyasu, Shinsaku Yata, Mikiya Kishino, Yuki Murakami, Yuji Shishido, Kozo Miyatani, Kyoichi Kihara, Tomoyuki Matsunaga, Manabu Yamamoto, Teruhisa Sakamoto, Toshimichi Hasegawa, Yoshiyuki Fujiwara

**Affiliations:** ^1^ Department of Surgery, Division of Gastrointestinal and Pediatric Surgery, School of Medicine, Faculty of Medicine Tottori University Yonago Japan; ^2^ Department of Multidisciplinary Internal Medicine, Division of Radiology, School of Medicine, Faculty of Medicine Tottori University Yonago Japan

**Keywords:** angioembolization, complications, hepatic necrosis, hepatic trauma

## Abstract

**Key Clinical Message:**

Although partial hepatic necrosis often occurs following endovascular treatment for bleeding associated with hepatic trauma, it is relatively rare that additional treatment is required. However, invasive procedures such as hepatic resection should sometimes be considered when infection occurs over massive hepatic necrosis.

**Abstract:**

Although partial hepatic necrosis following endovascular treatment for bleeding associated with hepatic trauma is occasionally experienced, it is relatively rare for the necrotic area of the liver to require additional treatment. However, invasive procedures such as hepatic resection should sometimes be considered when infection occurs over massive hepatic necrosis.

## Graphical abstract

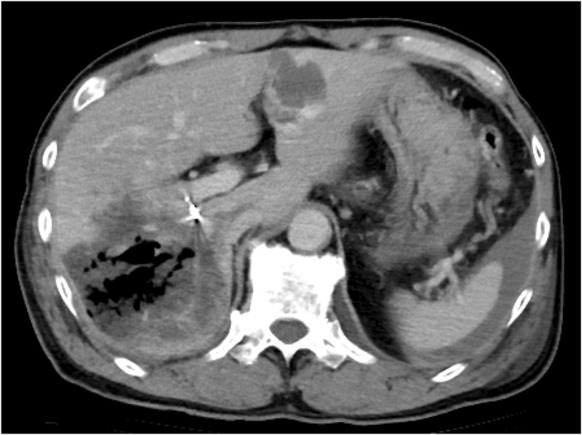

## 1 INTRODUCTION

Transcatheter arterial embolization (TAE) is increasingly used as the initial treatment of choice for hemorrhage associated with liver trauma.^1^ Small areas of liver necrosis following TAE are often noted, although complications such as infection rarely require additional procedures.^1^ When infection occurs in the necrotic area of the liver, the following options are available: (1) conservative treatment with antibiotics, (2) additional drainage, and (3) surgical resection or surgical debridement. If less invasive methods are not successful, surgical treatment must be considered. This report discusses a patient who required TAE hemostasis for trauma‐related posterior portal vein occlusion and arterial hemorrhage followed by right hepatectomy for massive hepatic necrosis (MHN) with infection.

## 2 CASE PRESENTATION

**FIGURE 1:**
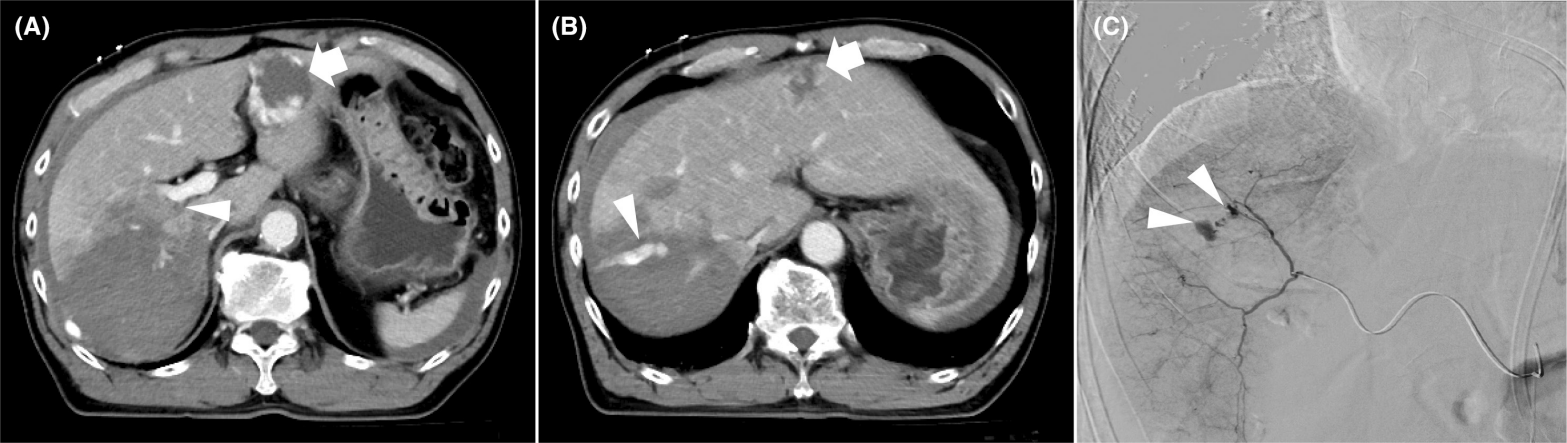
Imaging on the day of admission. (A) Contrast‐enhanced CT scan showing loss of contrast effect in the posterior section of the liver and occlusion of the posterior portal venous trunk (arrowhead). (B) Extravasation from the segment VII artery can be seen. Arrows indicate hemangiomas in segment IV, as noted initially (A, B). (C) A hepatic angiogram confirmed active segment VII artery bleeding (arrowhead). The bleeding artery was embolized.

The patient is a 57‐year‐old male injured in a collision between dump trucks who was transferred to the emergency room of our hospital. On arrival, vital signs included temperature, 36.4°C; blood pressure, 121/74 mmHg; heart rate, 78 bpm; respiratory rate, 24 breaths/min; and Glasgow coma scale score, 15. A focused ultrasound assessment for trauma noted an intraperitoneal cavity fluid collection. Contrast‐enhanced computed tomography (CT) noted grade IV liver damage^2^ and a loss of contrast effect in the posterior aspect of the liver. This was due to occlusion of the posterior branch of the portal vein and extravasation from the artery in liver segment VII (Figure 1A,B). The ischemic posterior section included approximately 36% of the total liver volume. In addition to the liver injury, an unstable fracture of the left iliac bone was noted, vital signs were relatively stable, and it was decided to proceed directly to interventional radiology. Angiography of the posterior branch of the hepatic artery noted extravasation from the artery in liver segment VII, and metal coil and gelatin sponge embolization of the artery were performed (Figure 1C).

**FIGURE 2:**
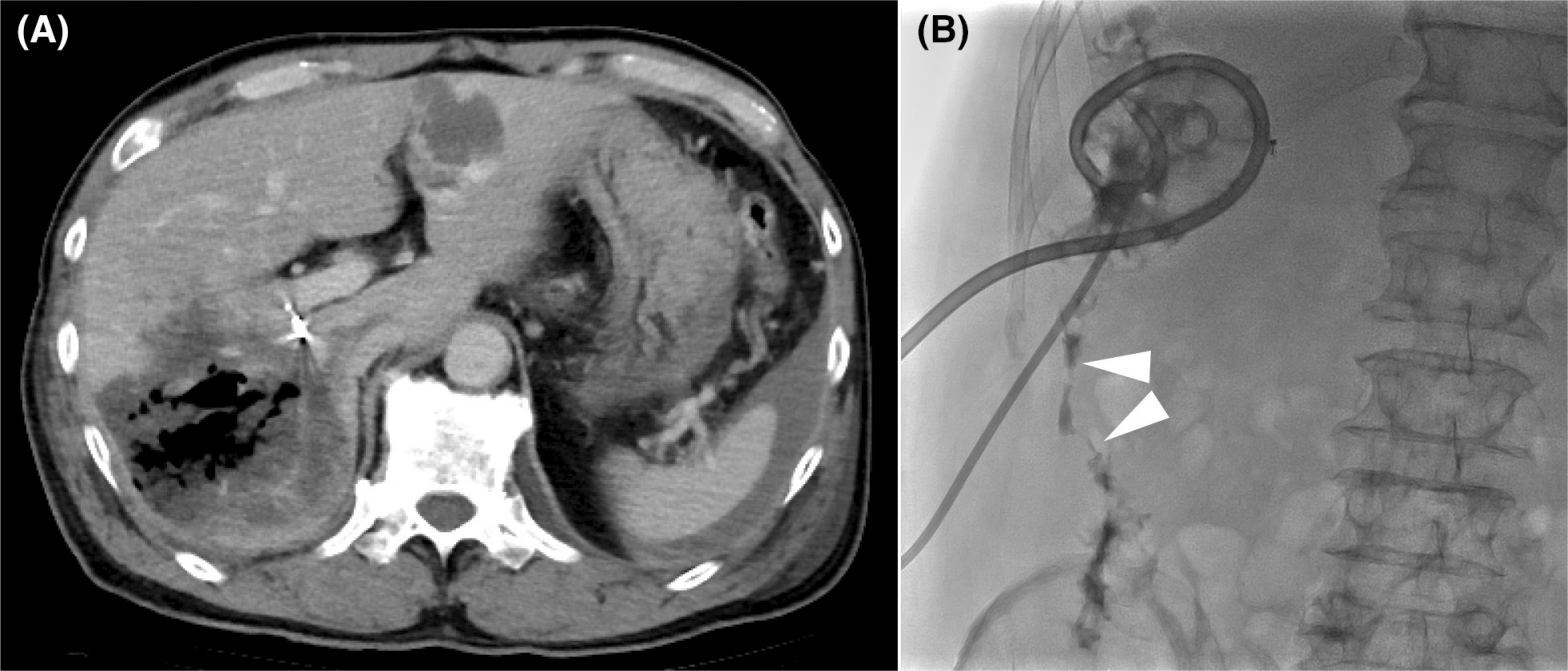
Images following the hemostatic procedure. (A) Computed tomography on the fifth hospital day. There was gas concentrated in the necrotic area of the liver, and infection originating from the bile duct was suspected. Note the presence of a hemostatic coil in the main trunk of the posterior arterial section. (B) Necrotic cavity imaging after placement of two drainage tubes in the area of necrosis. Fistula formation (arrowheads) from the necrotic area to the colon was proven.

Vital signs were stable following the intervention; however, on the fifth day of hospitalization, he suddenly developed a fever of 38°C. A CT scan revealed necrosis of the posterior section of the liver (Figure 2A). A blood culture was positive for *Clostridium butyricum*. It was determined that the bacteria had infected the area of liver necrosis via the bile duct.

Antibiotic therapy was immediately started. Although the signs of infection improved, the necrotic area did not shrink, and inflammatory findings worsened again after the antibiotics were terminated. Percutaneous CT‐guided drainage tubes were inserted on the 48th and 63rd days of hospitalization. Thick and purulent fluid continued to drain from both drains. The culture of the drainage fluid grew *Clostridium butyricum*, which was identical to the previous blood culture. The abscess cavity was flushed daily; however, imaging taken to adjust the drainage tube position noted a fistula in the ascending colon (Figure 2B).

**FIGURE 3:**
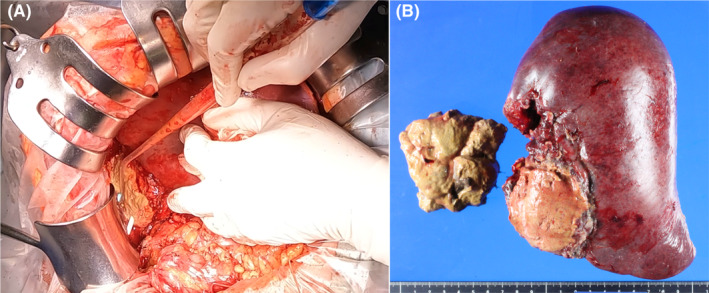
Operative findings. (A) Operative view of the right lobe. Two percutaneous drainage tubes inserted into the posterior section of the yellow necrotic liver can be seen. (B) Gross image of the resected right lobe. The posterior section consists of a brittle yellow necrotic substance.

Given concerns over a retrograde infection via the fistula, a right hepatectomy that included a necrotic area of the posterior section of the liver and fistula‐closing surgery were performed on the 72nd day of admission. Just prior to surgery, CT volumetry showed sufficient enlargement of the residual liver compared to the initial examination. The boundary between the left and right sides of the liver was mostly normal. Although a significant amount of adhesion between the dorsal portion of the necrotic site and the retroperitoneum (Figure 3A,B) made dissection difficult with increased hemorrhage, the hepatectomy was successfully performed. The fistula to the colon was suture closed without resection. The total operative time was 299 minutes. Although total blood loss was 780 mL, no blood transfusion was required peri‐operatively.

The patient underwent rehabilitation for a pelvic fracture and was discharged on the 28th postoperative day. This was a total of 100 days since his initial admission. Upon discharge, he was able to walk unaided with ease. The patient was seen 2 months postoperatively. Since he had experienced no further complications, his hospital follow‐up was complete.

## 3 DISCUSSION

Treatment strategies for liver injury associated with blunt abdominal trauma vary depending on the patient's condition.^3^ However, a non‐surgical, multidisciplinary approach in which surgeons, intensivists, and interventional radiologists work together to manage patients is essential. TAE with interventional radiology brought about a paradigm shift to conservative treatment of hemodynamically stable liver injuries with many reports of its usefulness.^4^, ^5^ Multidisciplinary approaches, including TAE, have improved mortality rates in patients with liver trauma. In patients with severe hepatic injury due to trauma, liver‐related complications have been reported in more than 50%.^6^ Therefore, complications following treatment of liver trauma must also be understood.^3^, ^7^

Because the liver is an organ with both an arterial and portal blood supply, ischemia from hemostatic procedures utilizing TAE is unlikely to be a problem. Still, in severe liver injuries, there can be significant tissue disruption and destruction of the major vasculature. TAE hemostasis for hemorrhage following complex liver injury can result in more MHN than might be imagined.^6^

Liver necrosis due to metabolic and inflammatory diseases is defined as submassive when necrosis accounts for 26%–75% of liver parenchymal volume, and MHN when necrosis accounts for more than 75%.^8^ However, MHN associated with trauma is ambiguously defined, and it is sometimes defined as hepatic ischemia requiring significant therapeutic intervention such as anatomic or non‐anatomic hepatectomy, debridement, and drainage.^7^ The definition of MHN related to trauma clearly differs from MHN caused by inflammatory or metabolic diseases. Small areas of hepatic necrosis following TAE for trauma are often experienced. Segalini et al., in their review of TAE hemostasis for liver trauma, reported that MHN occurred in 16% of cases.^1^

In this case, hepatic necrosis of the posterior section occurred after TAE hemostasis for hemorrhage associated with blunt hepatic trauma. Traumatic occlusion of the posterior portal vein appeared to be the reason for necrosis after TAE. Following drainage of the infectious foci of hepatic necrosis, fistula formation between the necrotic area and the colon became evident. Although there are reports of MHN resolution with continuous drainage and flushing,^9^, ^10^ hospitalization and prolonged treatment are worrisome issues. The patient was ultimately discharged from the hospital following right hepatic lobectomy. The 2 months' time after the initial injury and before surgery was long enough for the patient's condition to stabilize after multiple traumas and to confirm enlargement of the liver outside of the necrotic area. This allowed surgery to proceed without concern for postoperative liver failure. However, in retrospect, earlier surgical intervention might have been possible. According to Dobbs et al., when surgery is indicated, delaying surgery is not associated with improved survival.^7^ Still, more appropriate timing seemed challenging to achieve. In this case, there were, fortunately, no bile‐related problems during the patient's clinical course from the time of injury until the decision was made to resect the liver. If biliary leakage had become apparent during conservative treatment, the timing of surgery might have been accelerated. Although it may have taken longer to decide on liver resection, we believe that resection, including the necrotic area with infection, ultimately resulted in a shorter treatment time than continued drainage.

There are only a few case reports of HMN associated with liver trauma treated with hepatectomy.^11^, ^12^ Although the right lobectomy was successful in this case, surgical debridement of the necrotic area alone may have been sufficient to shorten the operative time and preserve liver function. Although some retrospective reports have compared debridement with hepatectomy,^1^ at this point there seems to be no evidence favoring either approach.

Although there are several guidelines for the hyper‐acute phase of liver trauma [6,15,16], there is no set treatment strategy for MHN from infection. This can be problematic in the subacute and late phases as was experienced in this case. Although it is difficult to determine the precise indication for surgical treatment of MHN following post‐traumatic TAE of the liver, hepatic resection may be indicated if complications including infection, bile leakage, or prolonged treatment period are anticipated.

## 4 CONCLUSION

In summary, this report describes a patient with hepatectomy of the right lobe of the liver for MHN from infection following TAE hemostasis for hemorrhage associated with hepatic trauma. The treatment strategy for MHN related to blunt liver trauma depends on the patient's general condition, the extent of other injuries, the degree of hepatic injury, and the clinical course following initial treatment. Therefore, it is necessary to closely observe the patient's condition and carefully decide on a treatment strategy.

## AUTHOR CONTRIBUTIONS

**Takehiko Hanaki:** Conceptualization; data curation; resources; writing – original draft. **Naruo Tokuyasu:** Writing – original draft. **Shinsaku Yata:** Resources; writing – review and editing. **Mikiya Kishino:** Writing – review and editing. **Yuki Murakami:** Writing – review and editing. **Yuji Shishido:** Writing – review and editing. **Kozo Miyatani:** Writing – review and editing. **Kyoichi Kihara:** Writing – review and editing. **Tomoyuki Matsunaga:** Writing – original draft. **Manabu Yamamoto:** Writing – original draft. **Teruhisa Sakamoto:** Supervision; writing – review and editing. **Toshimichi Hasegawa:** Supervision. **Yoshiyuki Fujiwara:** Supervision.

## FUNDING INFORMATION

This report received no specific grant from public, commercial, or not‐for‐profit funding agencies.

## CONFLICT OF INTEREST STATEMENT

The authors have no conflicts of interest or financial ties to disclose.

## CONSENT

Written informed consent was obtained from the patient to publish this case report and accompanying images. A copy of the written permission is available for review by this journal's editor in chief.

## Data Availability

The relevant data and images related to the patient's course and care are included in the article.
